# Genetic Mutation and Exosome Signature of Human Papilloma Virus Associated Oropharyngeal Cancer

**DOI:** 10.1038/srep46102

**Published:** 2017-04-06

**Authors:** Anbarasu Kannan, Kate L. Hertweck, Julie V. Philley, Robert B. Wells, Santanu Dasgupta

**Affiliations:** 1 Department of Cellular and Molecular Biology, The University of Texas Health Science Center at Tyler, Tyler, Texas, USA; 2 Department of Biology, The University of Texas at Tyler, Tyler, Texas, USA; 3 Department of Medicine, The University of Texas Health Science Center at Tyler, Tyler, Texas, USA; 4 Department of Pathology, The University of Texas Health Science Center at Tyler, Tyler, Texas., USA

## Abstract

Human papilloma virus-16 (HPV-16) associated oropharyngeal cancer (HPVOPC) is increasing alarmingly in the United States. We performed whole genome sequencing of a 44 year old, male HPVOPC subject diagnosed with moderately differentiated tonsillar carcinoma. We identified new somatic mutation in MUC16 (A.k.a. CA-125), MUC12, MUC4, MUC6, MUC2, SIRPA, HLA-DRB1, HLA-A and HLA-B molecules. Increased protein expression of MUC16, SIRPA and decreased expression of HLA-DRB1 was further demonstrated in this HPVOPC subject and an additional set of 15 HPVOPC cases. Copy number gain (3 copies) was also observed for MUC2, MUC4, MUC6 and SIRPA. Enhanced expression of MUC16, SIRPA and HPV-16-E7 protein was detectable in the circulating exosomes of numerous HPVOPC subjects. Treatment of non-tumorigenic mammary epithelial cells with exosomes derived from aggressive HPVOPC cells harboring MUC16, SIRPA and HPV-16-E7 proteins augmented invasion and induced epithelial to mesenchymal transition (EMT) accompanied by an increased expression ratio of the EMT markers Vimentin/E-cadherin. Exosome based screening of key HPVOPC associated molecules could be beneficial for early cancer diagnosis, monitoring and surveillance.

Human papilloma virus-16 (HPV-16) associated oropharyngeal cancer (HPVOPC) is becoming epidemic in the United States^1^,^2^,^3^,^4^,^5^,^6^,^7^,^8^,^9^,^10^. Human papilloma virus induced tumorigenesis occurs primarily in the oropharynx region (tonsil or base of tongue), where HPV derived oncoproteins E6 and E7 inactivate p53 and retinoblastoma (Rb), resulting in transformation and eventual progression to malignancy^2^,^3^. A significant proportion of the HPVOPCs have a high rate of loco-regional recurrence and poor clinical outcome^4^,^6^,^10^. Given the limitation of HPV alone as a significant predictor of advanced HPVOPC^2^, development of additional prognostic markers would be critical to reduce morbidity and mortality. Next generation sequencing (NGS) has emerged as a powerful tool to identify molecular changes associated with disease development and progression^11^,^12^,^13^,^14^,^15^,^16^ and could be invaluable for developing early detection, surveillance and therapeutic strategies for various malignancies.

Exosomes are biologically active, small (50–200 nm) endocytic vesicles present in all cell types, circulation and body fluids and enriched in specific nucleic acids and proteins^17^,^18^,^19^,^20^,^21^. The exosomes are regarded as a new pathway for regulating favorable micro-environment by the malignant and virus infected cells^22^. Exosomes can also act in disseminating infection and immune evasion^22^ and bear immense potential for biomarker and therapeutic development.

Human Papilloma Virus is a well-known risk factor for malignant transformation and increasingly associated with the majority (60–70%) of the recently diagnosed oropharyngeal cancer incidences^22^. However to date, very few exosome associated studies have been conducted on HPV-associated oropharyngeal cancers^22^. With the increasing evidences on the role of the exosomes in viral infection and intercellular communications, the reservoir of these exosomes in HPV-associated cancers definitely warrant further interrogation^22^.

In this study, whole genome sequencing analysis of a HPVOPC patient uncovered new somatic mutation, copy number gain and aberrant protein expression in various molecules, not well defined in HPVOPCs. Expression of the key HPVOPC associated molecules, including MUC16, SIRPA accompanied by HPV-16-E7 protein was detected in the circulating exosomes of some HPVOPC patients. The exosomes secreted from aggressive HPVOPC cells harboring MUC16, SIRPA and HPV-16-E7 proteins enhanced invasion and induced epithelial to mesenchymal transition of non-tumorigenic mammary epithelial cells.

## Result

### Spectrum of genome-wide somatic mutations in the HPVOPC patient

**Figure 1:**
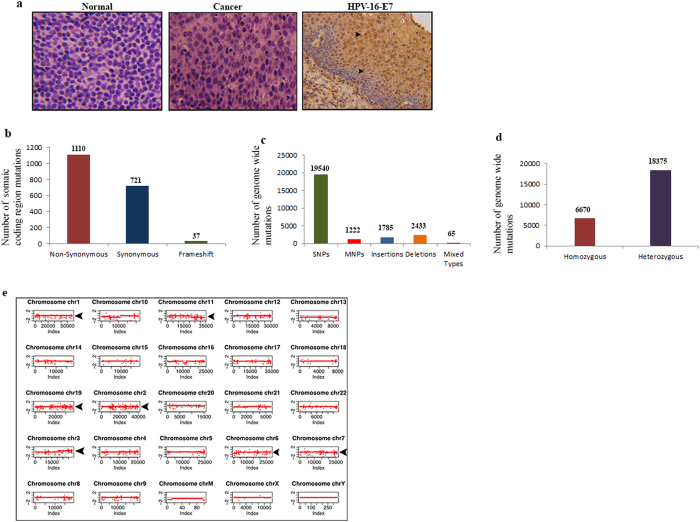
Tissue histology, HPV and mutation detection. (**a**) Histopathological analysis of the normal and cancer tissues by Hematoxylene and Eosine staining. HPV-16-E7 protein was detected (arrowheads) by immunohistochemistry using a specific antibody as described under the material and method section (**a**). Image magnification X 200. (**b**) The total number of somatic non-synonymous, synonymous and frameshift mutations from the coding regions of the HPVOPC subject. (**c**) The overall number of somatic mutations covering the coding and non coding regions of the genome. (**d**) Number of homozygous and heterozygous mutations observed spanning the entire genome. (**e**) High copy number variation in the chromosomal regions 1, 2, 3, 6, 7, 11 and 19 (arrowheads).

In this study, we conducted a whole genome NGS analysis of paired normal and cancer tissues obtained from a 44 year old, male HPVOPC patient, diagnosed with moderately differentiated tonsillar squamous cell carcinoma (Fig. 1a) on the Illumina’s Hiseq sequencing platform. Considering only coding regions, we observed 1110 somatic non-synonymous, 721 synonymous and 37 frame shift mutations (Total = 1868) in this patient (Fig. 1b). Genome wide, the total number of mutations were 25045 (Fig. 1c). These mutations include SNPs (N = 19540), MNPs (N = 1222), insertions (N = 1785), deletions (N = 2433) and mixed types (N = 65) (Fig. 1c). In terms of zygosity covering the whole genome, we observed 6670 homozygous and 18,375 heterozygous mutations (N = 25045) (Fig. 1d). Globally, considerable copy number variation was observed in various chromosomal regions, including chromosome 1, 2, 6, 7, 11 and 19 (Fig. 1e).

### Frequently mutated genes and their copy number variation in the HPVOPC patient

**Figure 2:**
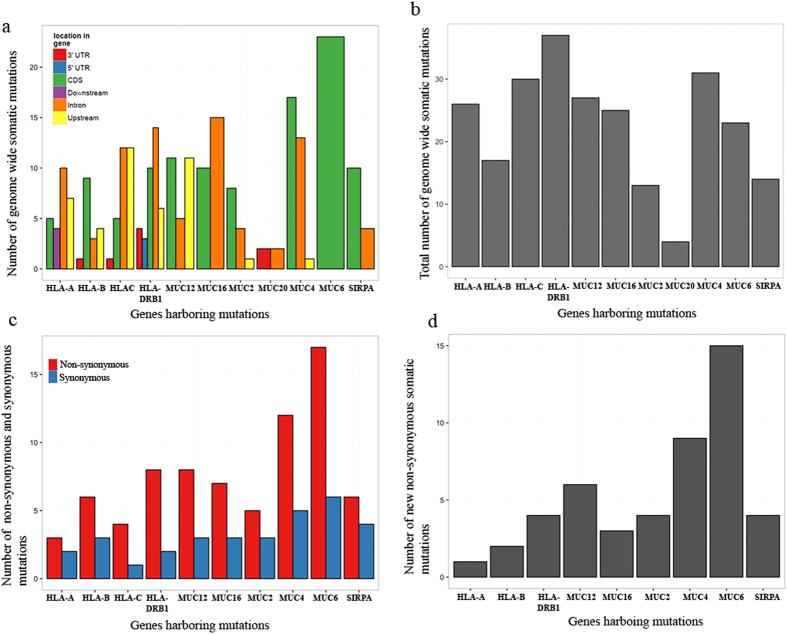
Genome wide mutation spectrum of the HPVOPC patient. (**a**) List of genes with the highest number of somatic mutations spanning various locations as indicated. The colored boxes represent the location of the genomic region, where the mutations were observed. (**b**) Total number of genome wide somatic mutations for each gene listed in (**a**). (**c**) The extent of non-synonymous (red box) and synonymous (blue box) coding region mutations in the gene panel. (**d**) New non-synonymous somatic mutations in the most frequently altered gene panel (Table 1). UTR: Untranslated region; CDS; coding DNA sequence.

The highest frequency of somatic mutations spanning the coding regions (CDS) was observed in a panel of genes involved in various pathways associated with malignant progression^23^,^24^,^25^,^26^,^27^,^28^,^29^. These molecules include MUC16 (A.k.a. CA-125), MUC12, MUC4, MUC6, MUC2, SIRPA, HLA-DRB1, HLA-A and HLA-B (Table 1 and Fig. 2). The majority of the novel mutations observed in these genes was missense in nature except for 1 frameshift mutation noted in MUC12 and SIRPA (Table 1). Among these molecules, MUC16 (chr.19) had 2 missense mutations, MUC12 (Chr. 7) had 4 missense, 1 frameshift, MUC4 (chr. 3) had 7 missense, MUC6 (chr.11) had 11 missense, MUC2 had 4 missense, SIRPA had 3 missense and 1 frameshift, HLA-DRB1 (chr. 6) had 4 missense, HLA-A (chr. 6) had 1 missense and HLA-B (chr. 6) had 2 missense mutations (Table 1). Other than the CDS, mutations in these molecules were observed in various other regions, including the 5′UTR, 3′UTR, introns as well as their upstream and downstream regions (Fig. 2a,b). The majority of the CDS mutations in these molecules was non-synonymous in nature (Fig. 2c,d) and only novel mutations were represented in Fig. 2c,d and Table 1. Notably, we observed copy number gain (3 copies) for MUC2, MUC4, MUC6 and SIRPA (Table 1).

### Association between somatic mutation and corresponding protein expression in the HPVOPC patient

**Figure 3:**
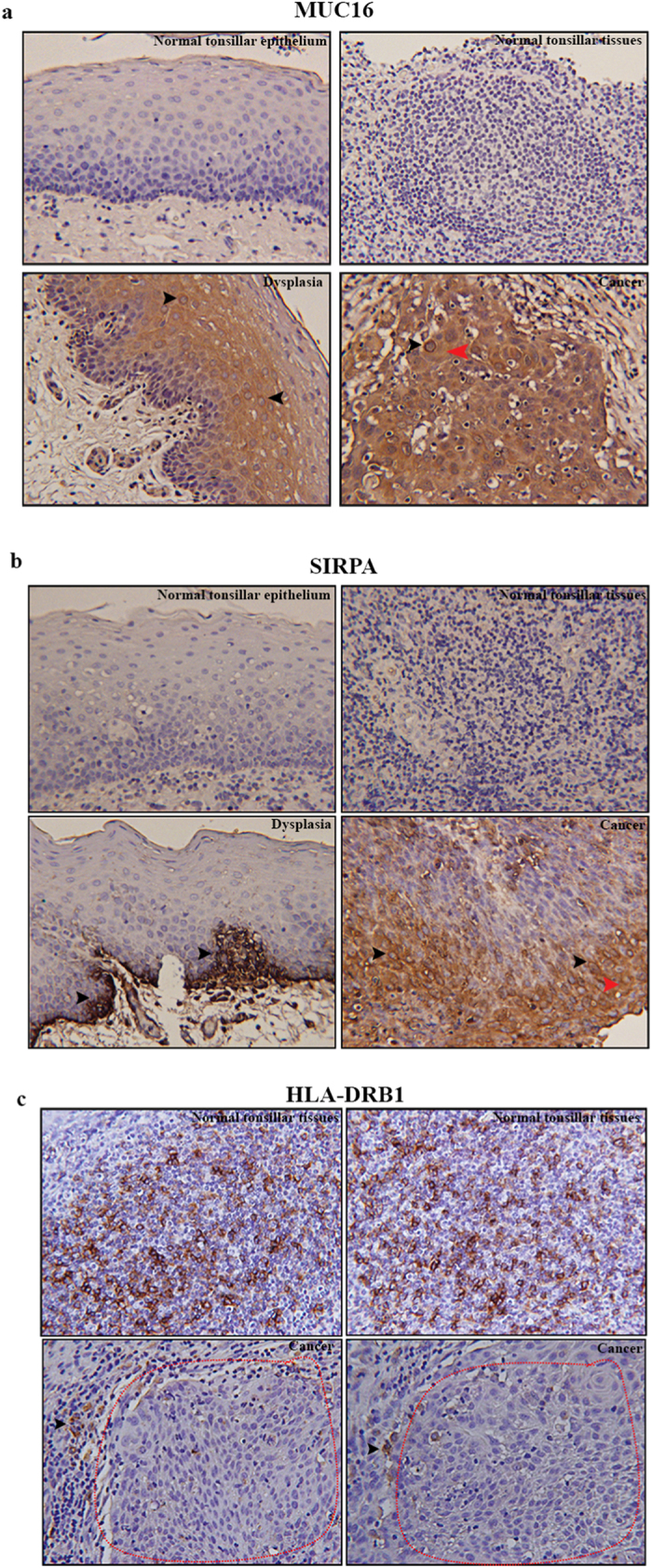
Expression pattern of MUC16, SIRPA and HLA-DRB1 in the primary HPVOPC tissues. Compared to the matched normal counterparts, high expression of MUC16 (**a**) and SIRPA (**b**) in both the dysplasia and cancer tissues (arrowheads). The expression of MUC16 in the cancer tissues was both membrane bound (black arrowhead) and secretory (red arrowhead). The expression of HLA-DRB1 was barely detectable (p = 0.0002) in the cancerous tissues (encircled) compared to the matched normal tonsillar tissues (**c**). Image magnification X 200.

To examine the corresponding protein expression pattern of some of the genes exhibiting somatic mutations, we performed immunohistochemistry (IHC) using antibodies specific for MUC16, SIRPA and HLA-DRB1 on paired normal and malignant FFPE tissue samples obtained from this HPVOPC subject. We observed an exclusively abundant expression of MUC16 and SIRPA in the dysplastic lesions and the corresponding carcinoma tissues of this patient (Fig. 3a,b). The expression of MUC16 and SIRPA was barely detectable in the cancer adjacent normal tissues (Fig. 3a,b). The expression pattern of MUC16 and SIRPA appears to be both membrane bound (black arrowheads) and secretory (red arrowheads) (Fig. 3a,b). On the other hand, marked loss (p = 0.0002) of HLA-DRB1 expression was evident in the tumor tissues compared to the normal tonsillar tissues (Fig. 3c). Appreciable expression of HLA-DRB1 was evident in tumor adjacent normal appearing tonsillar tissues (Fig. 3c, black arrowheads).

### Validation of the altered expression signature in HPVOPC cases

**Figure 4:**
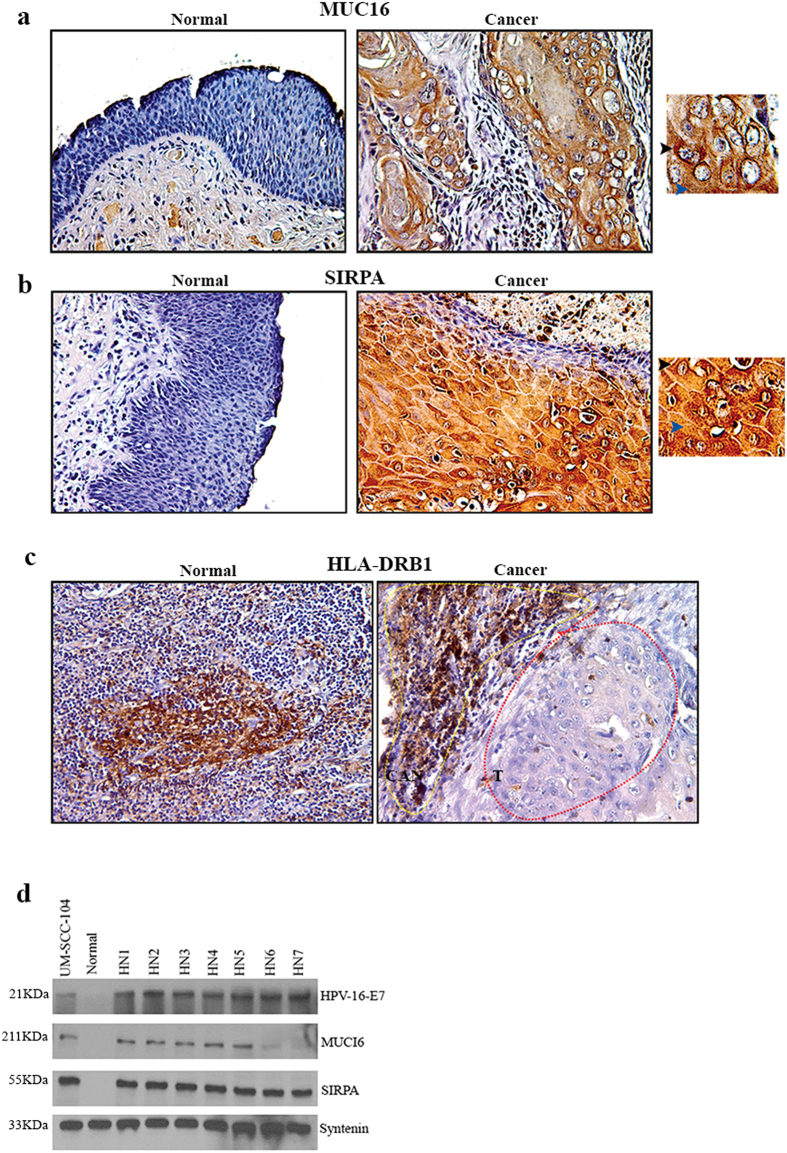
Expression pattern of MUC-16, SIRPA and HLA-DRB1 in HPVOPC subjects and the circulating exosomes. The expression of MUC-16 (**a**, p = 0.002–0.0001) and SIRPA (**b**, p = 0.003–0.0002) protein was higher in the cancer tissues compared to the matched normal tissues. The adjacent insets show membrane bound expression of both the proteins. The expression of HLA-DRB1 was barely detectable in the cancerous tissues (encircled, red) compared to the matched normal (**c**, p = 0.003–0.004). High HLA-DRB1 expression was also detectable in the cancer adjacent normal (CAN) tissues (encircled, yellow). Image magnification X 200. (**d**) Detection of HPV-16-E7, MUC-16 and SIRPA expression in the circulating exosomes of 7 additional HPVOPC cases (HN1-HN7). The expression of HPV-16-E7, MUC-16 and SIRPA was undetectable in the circulating exosomes obtained from cancer-free healthy individual (**d**). UM-SCC-104, a HPVOPC patient derived aggressive cell line was used as a positive control for HPV-16-E7 integration. Syntenin was used as an exosome marker.

We observed increased expression of MUC16, SIRPA and decreased expression of HLA-DRB1 in the HPVOPC patient harboring somatic mutations in these molecules. To further examine the expression pattern of these molecules, we performed IHC for MUC16, SIRPA and HLA-DRB1 in an additional set of 15 HPVOPC cases. We observed increased expression (p = 0.002–0.0001) of MUC16 in 73% (11/15) cases and SIRPA expression (p = 0.003–0.0002) in 80% (12/15) of the HPVPOC cases compared to their matched normal tissues (Fig. 4a,b). Similar to the HPVOPC subject we analyzed for MUC16 and SIRPA expression (Fig. 3), the expression pattern of these proteins were both membrane bound (black arrowheads) and secretory (blue arrowheads) in the additional HPVOPC cases (Fig. 4). On the other hand, loss of HLA-DRB1 expression (p = 0.003–0.004) was observed in 86% (13/15) of the HPVOPC cases compared to the adjacent normal tissues (Fig. 4c). The carcinoma (encircled in red) surrounding normal appearing tonsillar tissues (encircled in yellow) exhibited appreciable amount of HLA-DRB1 expression (Fig. 4c).

### Detection of HPV-16 and key altered proteins in the circulating exosomes of the HPVOPC patients

Exosomes play a critical role in cellular transformation and infection^17^,^18^,^19^,^20^,^21^. To determine whether the circulating exosomes of the HPVOPC subjects carry the abundantly expressed proteins MUC16 and SIRPA, we evaluated sera exosomes purified from 7 HPVOPC patients. We confirmed the presence of HPV-16-E7 and SIRPA proteins in 100% (7/7) of the subjects (Fig. 4d). We also observed abundant expression of MUC16 in the sera exosomes of 71% (5/7) of the HPVOPC cases (Fig. 4d).

### Effect of HPVOPC cells’ secreted exosomes on epithelial to mesenchymal transition and invasion

**Figure 5:**
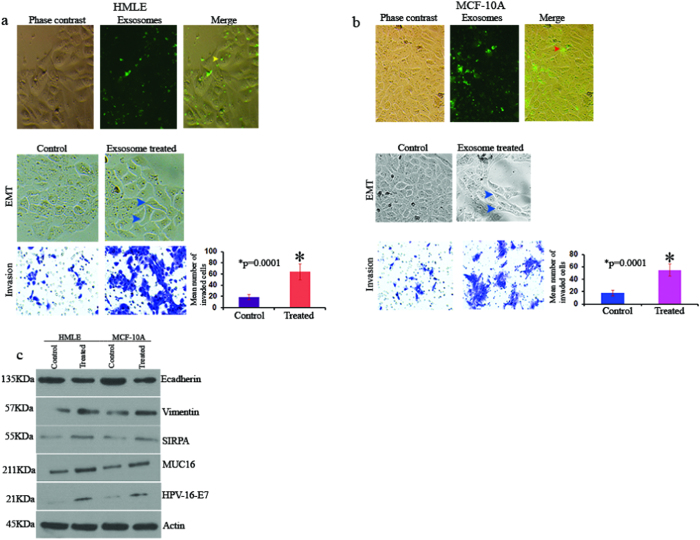
Impact of exosomes treatment on EMT and invasion. (**a**) Confirmation of the exosome uptake (yellow arrowheads, upper panel) by the HMLE cells. Increased invasion (p = 0.0001) and induction of EMT phenotype (arrowheads) in the exosome treated HMLE cells. (**b**) Confirmation of the exosome uptake (red arrowheads, upper panel) by the MCF-10A cells. Enhanced invasion (p = 0.0001) and modulation of EMT phenotype (arrowheads) in the exosome treated MCF-10A cells. (**c**) Detection of increased Vimentin/E-cadharin expression ratio (panel 1 and 2) accompanied by enhanced expression of SIRPA and MUC16 (panel 3 and 4) in the cells treated with UM-SCC-104 cells’ secreted exosomes. Detection of HPV-16-E7 protein (panel 5) in the exosome treated cells (**c**). Actin was used as a loading control. Magnification X 200 (**a**,**b**). Control: cells treated with PBS; Treated: cells treated with UM-SCC-104 cells’ secreted exosomes.

The UM-SCC-104 cell line used in this study was generated from a recurrent HPVOPC patient^30^ and the secreted exosomes from these cells were found to harbor HPV-16-E7, MUC16 and SIRPA proteins (Fig. 4d). To determine the impact of these exosomes on influencing cell growth, we co-cultured UM-SCC-104 secreted exosomes with a couple of non-tumorigenic and HPV-16^−^ human mammary epithelial cell lines for 1 week and measured epithelial to mesenchymal transition (EMT) changes and invasion potential of these epithelial cells. First, we confirmed the entry of the exosomes in these cells (Fig. 5a,b arrowheads). We observed an EMT phenotype (arrowheads) as determined by cell morphology^31^,^32^ accompanied by an increased invasion (p = 0.0001) of both the cell lines following the exosomes treatment (Fig. 5a,b). In addition, an increased expression ratio of the EMT markers Vimentin/E-cadherin was observed in these exosome treated cells accompanied by an enhanced expression of SIRPA and MUC16 (Fig. 5c). Notably, these mammary epithelial cells were also found to harbor HPV-16-E7 protein following the exosome treatment (Fig. 5c).

## Discussion

Human papilloma virus is well regarded as a powerful microbe that can drive tumorigenesis by interacting with the human genome^33^. The HPVOPC is an HPV-16-associated, rapidly increasing malignancy in the USA and worldwide, particularly among the young adults^1^,^2^,^3^,^4^,^5^,^6^,^7^,^8^,^9^,^10^. Understanding the molecular pathogenesis of HPVOPC progression is critical for developing better disease management strategies.

Characterization of the pathogenic genomic alteration through NGS in various malignancies can be a powerful tool. At the same time, determining the frequency and impact of such alterations on patients is also necessary to evaluate disease development and progression. In this study, we identified numerous mutations in the HPVOPC subject from the mucin family members, including MUC2, MUC4, MUC6, MUC12 and MUC16, a well known marker for ovarian cancer^34^. Mutations in MUC16 were associated with abundant expression during HPVOPC progression. These results suggest for a potential role for MUC16 in promoting cancer through the disruption of epithelial cell polarity and facilitating EMT^23^. Possibly as a reason, HPVOPC cells’ derived exosomes enriched in MUC16 protein induced EMT and increased invasion of the non-tumorigenic mammary epithelial cells accompanied by an increased expression ratio of the well known EMT markers. Robust expression of MUC16 observed in additional HPVOPC subjects suggests for its potential role in HPVOPC tumorigenesis. However, the mutation spectrum of MUC16 in these cases is unknown. Other NGS studies in HPV^−^ and HPV^+^ head and neck cancer have reported mutations in MUC16 and other mucins (e.g., MUC4, MUC12 and MUC6)^12^,^23^. Our study identified new mutations in these molecules and also discovered increased copy number of MUC2, MUC4 and MUC6. A few other studies have reported an association between elevated MUC16 level and progression of oral squamous cell carcinomas and thyroid neoplasm^27^,^28^,^29^. In addition to the mucins, SIRPA displayed somatic mutations, copy number gain and corresponding overexpression in the progressive stages of this subject and the majority of the additional HPVOPC subjects analyzed. SIRPA has been implicated as a potential therapeutic target in human malignancies^24^, although its expression pattern and role in HPVOPC remains obscured. Thus, MUC16 and SIRPA could be potential targets for biomarker and therapeutic development for HPVOPC and a large scale analysis of these molecules are warranted.

Immunosuppression plays a critical role during HPVOPC evolution^35^, which could be developed through an interplay between the viral genome and immunoregulatory molecules. Both human leukocyte antigens I and II (HLA-I/II) play an important role in host immune responses to cancer cells and their aberrant expression might potentially drive cancer initiation and subsequent progression^25^,^26^. Therefore, mutations and corresponding loss of expression of HLA-DRB1 along with mutations in HLA-A/B might be associated with the development of an immunosuppressive microenvironment necessary for HPVOPC progression.

Exosomes are emerging as the mediators of malignant transformation, immunomodulation, and utilization by human tumor virus for intercellular communications through selective transport of nucleic acids and proteins^17^,^18^,^19^,^20^,^21^,^36^. Perhaps the most striking finding of our study is the abundance of the key altered proteins MUC16, SIRPA and the HPV-16-E7 in the circulating exosomes of the HPVOPC subjects, which might have facilitated disease progression in concert with other molecules. Notably, the HPVOPC cells’ derived exosomes not only harbored the HPV-16-E7 protein along with MUC16 and SIRPA but also successfully transported HPV-16-E7 in the HPV^−^ mammary epithelial cells. On the other hand, increased expression of MUC16 and SIRPA observed in these cells following exosome treatment could possibly be due to their transport through the exosomes. Thus, exosomes might be a feasible means for sustained infection and subsequent tumorigenic progression.

Our study suggests a possible association between genomic alterations and aberrant expression of corresponding protein in the progressive development of HPVOPC. Key altered proteins and HPV-16 were detected in the circulating exosomes as well. Monitoring HPVOPC associated viral load and key molecular changes in the circulating exosomes bears potential to develop suitable disease management strategies.

## Materials and Methods

### Archived human tissue samples and ethical statement

Fresh frozen archived cancer and adjacent normal tissues were obtained from a 44 year old male, HPV-16 positive patient diagnosed with moderately differentiated squamous cell carcinoma of the right tonsil (T1N1M0). The patient underwent tonsillectomy with adenoidectomy and received chemoradiation (XRT-70Gy, Cisplatin- 3 cycles) following the surgery. The surgical specimens were collected before any treatment. We also collected paraffin embedded formalin fixed (FFPE) paired normal and cancer tissues from the above subject. Paraffin embedded formalin fixed paired normal and cancer tissues from an additional set of 15 HPVOPC cases were also collected from The University of Texas Health Science Center at Tyler (UTHCT). In addition, archived serum samples from 7 HPV-16 positive cases were collected from the Cooperative Human Tissue Network (CHTN). All archived samples were collected under IRB approved protocols. All the patients are de-identified. Only relevant clinical information such as age, grade, stage, diagnosis, HPV status, etc. were collected as per our IRB approved protocols. All samples were collected and archived by the Institutions with informed consent from all the subjects. All methods were performed in accordance with the relevant guidelines and regulations.

### Antibodies, cell lines and reagents

The MUC16 (#X325) and HPV-16-E7 (#ab20191) antibodies were purchased from Abcam. The HLA-DRB1 antibody (#LS-C138934) was obtained from LS Biosciences. The Syntenin antibody (#H00006386) was purchased from Abnova and SIRPA antibody (#PA5-29544) from Thermoscientific. The HPV-16-L1 antibody (#NB120-3199) was obtained from Novus Biological. Anti-rabbit E-cadherin (#24E10) and Vimentin (#D21H3) antibodies were purchased from Cell Signaling Inc. Anti-mouse (#115-035-003) and rabbit (#111-035-003) secondary antibodies were obtained from Jackson Immunoresearch. The HMLE cells ware kindly provided by Dr. Guojun Wu, Wayne State University and the MCF-10A cells were obtained from ATCC. The HPV-16 integrated UM-SCC-104 cell line generated from a recurrent HPVOPC subject^30^ was obtained from Dr. Thomas Carey, The University of Michigan Comprehensive Cancer Center. All cell lines were authenticated and periodically checked for mycoplasmal contamination using a mycoplasma detection kit (Sigma #MP-0025).

### Next Generation whole genome sequencing

Genomic DNA (gDNA) was extracted from fresh-frozen normal and tumor tissues^37^ and subjected to whole genome sequencing. Briefly, gDNA was subjected to agarose gel and OD ratio tests to confirm the purity and concentration prior to Bioruptor (Diagenode, Inc., Denville, NJ USA) fragmentation. Fragmented gDNAs were tested for size distribution and concentration using an Agilent Tapestation 2200 and Nanodrop. Illumina libraries were then made from qualified fragmented gDNA using NEBNext Library Prep MasterMix for Illumina (NEB, #E6040L) and the resulting libraries were subjected to exome enrichment using SureSelect AV4 Exon Coverage (Agilent Technologies, Wilmington, DE USA, #5190-4633) following manufacturer’s instructions. Enriched libraries were tested for enrichment by qPCR and for size distribution and concentration by an Agilent Bioanalyzer 2100. The samples were then sequenced on Illumina HiSeq 2000, using High Output v3 chemistry which generated paired-end reads of 101 nucleotides (nt).

### Data analysis

Data were analyzed for quality assessment using FASTQC (Babraham Institute, Cambridge, UK). We analyzed exome coverage, and exome-wide SNP/InDel using the platform provided by DNAnexus (DNAnexus, Inc, Mountain View, CA USA). Sequence reads were aligned to the mm10 reference genome with Burrows-Wheeler transform (BWA)^38^, followed by Picard (http://broadinstitute.github.io/picard) duplicates removal, GATK base quality score recalibration, and indel realignment^39^. SNP and INDEL discovery and genotyping of all samples was performed simultaneously with GATK Lite 2.3.9^40^,^41^. SNPs and indels were annotated with snpEff^42^. Variants from the carcinoma sample were filtered with the normal sample to remove all variants existing in the germ line (prior to tumor formation). Remaining variants were assumed to represent somatic mutations, and were classified by mutation type and location in the gene. We extracted nonsynonymous variants from the coding regions of target genes, and removed any variants previously reported in dbSNP. The sequencing data have been submitted to the NCBI Sequence Read Archive (SRA) and can be found under accession # SRP081299. Scripts used to parse and visualize data (including annotated SNP tables) can be found at https://github.com/k8hertweck/HNSCCtargetGenes.

### Immunohistochemistry and western blotting

Immunohistochemistry was performed using specific antibodies and conditions in paired primary tissue specimens (normal/tumor) as described earlier^31^,^32^. For comparison, all sections were processed in parallel. We used 1:50 dilution of primary antibodies and 1:250 for secondary antibodies in these analyses. At least 10-fields were randomly selected for examining the staining intensity and the distribution pattern of the proteins^31^,^32^. Western blotting analysis was performed as described earlier^31^,^32^.

### Exosome preparation from human sera

Exosomes were isolated from human sera using commercially available kits and protocols followed by protein isolation and Western blotting^32^,^43^ with Syntenin used as an exosome marker as previously described^32^,^43^.

### Exosome co-culture, EMT and invasion assays

Non-tumorigenic breast epithelial cells HMLE and MCF-10A were co-cultured with the exosomes (1 × 10^6^) isolated from the HPV-16 containing UM-SCC-104 cell line^30^ for 7 days followed by EMT assay and analysis as described earlier^31^,^32^,^43^. PBS was used as a control. Cell invasion (in triplicate wells) was assessed using the Cell Invasion Assay Kit (#354481, Corning)^31^,^32^. The cells were cultured in exosome depleted specific medium and the exosomes were pre-labeled for monitoring their uptake by the cells as described earlier^32^,^43^. In all cases, data were presented as mean ± SE of duplicate experiments.

### Statistical Analysis

We employed Chi-square, Fisher’s exact or Student’s *t* tests as appropriate. All p-values were two-sided and all confidence intervals were at the 95% level. Computation for all the analyses was performed using the Statistical Analysis System (SAS).

## Additional Information

**How to cite this article:** Kannan, A. *et al*. Genetic Mutation and Exosome Signature of Human Papilloma Virus Associated Oropharyngeal Cancer. *Sci. Rep.* **7**, 46102; doi: 10.1038/srep46102 (2017).

**Publisher's note:** Springer Nature remains neutral with regard to jurisdictional claims in published maps and institutional affiliations.

## Figures and Tables

**Table 1:** List of genes with frequent somatic mutation and copy number variation in the HPVOPC patient.

Gene	Chromosome	Position	Amino Acid Alteration	Type	Copy Number Variation
MUC16	Chr 19	8999446	S −> G	Missense	0
MUC16	Chr 19	8999479	Q −> K	Missense	0
MUC12	Chr 7	100612958	− −> A	***Frameshift***	0
MUC12	Chr 7	100637547	A −> T	Missense	0
MUC12	Chr 7	100641798	P −> A	Missense	0
MUC12	Chr 7	100643031	L −> I	Missense	0
MUC12	Chr 7	100646322	P −> T	Missense	0
MUC4	Chr 3	195508336	G −> D	Missense	**3**
MUC4	Chr 3	195508343	S −> T	Missense	**3**
MUC4	Chr 3	195508402	T −> N	Missense	**3**
MUC4	Chr 3	195509676	Q −> H	Missense	**3**
MUC4	Chr 3	195509717	P −> T	Missense	**3**
MUC4	Chr 3	195518101	D −> A	Missense	**3**
MUC4	Chr 3	195518103	P −> Q	Missense	**3**
MUC6	Chr 11	1016604	TV −> TP	Missense	**3**
MUC6	Chr 11	1016628	T −> I	Missense	**3**
MUC6	Chr 11	1016662	P −> S	Missense	**3**
MUC6	Chr 11	1016889	P −> S	Missense	**3**
MUC6	Chr 11	1017135	T −> I	Missense	**3**
MUC6	Chr 11	1017273	F −> S	Missense	**3**
MUC6	Chr 11	1017307	P −> S	Missense	**3**
MUC6	Chr 11	1017522	E −> A	Missense	**3**
MUC6	Chr 11	1017529	H −> Y	Missense	**3**
MUC6	Chr 11	1017912	T −> I	Missense	**3**
MUC6	Chr 11	1018459	P −> T	Missense	**3**
MUC2	Chr 11	1092715	A −> T	Missense	**3**
MUC2	Chr 11	1092928	S −> T	Missense	**3**
MUC2	Chr 11	1092941	T −> S	Missense	**3**
MUC2	Chr 11	1093057	T −> A	Missense	**3**
SIRPA	Chr 20	1895796	L −> S	Missense	**3**
SIRPA	Chr 20	1895950	DL −> ES	Missense	**3**
SIRPA	Chr 20	1896052	PD −> P	***Frameshift***	**3**
SIRPA	Chr 20	1896059	V −> T	Missense	**3**
HLA-DRB1	Chr 6	32548544	FI −> FL	Missense	0
HLA-DRB1	Chr 6	32551911	V −> G	Missense	0
HLA-DRB1	Chr 6	32551957	A −> K	Missense	0
HLA-DRB1	Chr 6	32552129	RE −> HE	Missense	0
HLA-A	Chr 6	29910716	Q −> G	Missense	0
HLA-B	Chr 6	31324200	S −> R	Missense	0
HLA-B	Chr 6	31324602	E −> M	Missense	0
